# Microglia and Astrocytes in Alzheimer’s Disease in the Context of the Aberrant Copper Homeostasis Hypothesis

**DOI:** 10.3390/biom11111598

**Published:** 2021-10-28

**Authors:** Amit Pal, Isha Rani, Anil Pawar, Mario Picozza, Mauro Rongioletti, Rosanna Squitti

**Affiliations:** 1 Department of Biochemistry, AIIMS, Kalyani 741245, West Bengal, India; 2 Department of Biochemistry, Maharishi Markandeshwar Institute of Medical Sciences and Research (MMIMSR), Maharishi Markandeshwar University (MMU), Mullana, Ambala 133207, Haryana, India; singlaisha8@gmail.com; 3 Department of Zoology, DAV University, Jalandhar 144012, Punjab, India; sumanil27@yahoo.co.in; 4 Neuroimmunology Unit, IRCSS Fondazione Santa Lucia, 00143 Rome, Italy; m.picozza@hsantalucia.it; 5 Department of Laboratory Medicine, Research and Development Division, San Giovanni Calibita Fatebenefratelli Hospital, Isola Tiberina, 00186 Rome, Italy; maurociroantonio.rongioletti@fbf-isola.it; 6 Molecular Markers Laboratory, IRCCS Istituto Centro San Giovanni di Dio Fatebenefratelli, 25125 Brescia, Italy

**Keywords:** astrocyte, microglia, copper, Alzheimer’s disease

## Abstract

Evidence of copper’s (Cu) involvement in Alzheimer’s disease (AD) is available, but information on Cu involvement in microglia and astrocytes during the course of AD has yet to be structurally discussed. This review deals with this matter in an attempt to provide an updated discussion on the role of reactive glia challenged by excess labile Cu in a wide picture that embraces all the major processes identified as playing a role in toxicity induced by an imbalance of Cu in AD.

## 1. Introduction

The pleiotropic pathogenesis of Alzheimer’s disease (AD), along with drugs developed in the “inside-out approach,” which relied heavily around halting/clearing intracellular aggregates of the hyper phosphorylated tau protein and extracellular plaques of the β-amyloid (Aβ) peptide, led to the failure of most clinical trials despite decades of research. Lately, thanks to the rapid development and improvement of imaging technologies and experimental research, attention has been diverted beyond the “classical neuronal death or β-amyloid cascade hypothesis” to the immediate surroundings around neurons, i.e., non-neuronal cells (glial cells) in neuronal health, aging, and AD (the outside-in approach) [1,2]. 

Among glial cells, astrocytes have rapidly emerged as cells with an important supporting role in learning and memory consolidation [3], along with protecting the central nervous system (CNS) from metal toxicity [4], including copper (Cu) neurotoxicity [5]. Microglia involvement is also increasingly factored into sporadic AD pathogenesis [6,7]. It has also been postulated that cognitive decline managed by modifiable lifestyle factors is thought to be mediated by the mitigation of aberrant microglia activation in aging and the subsequent suppression of neuroinflammation [8]. Intriguingly, CNS Cu dyshomeostasis hypothesis fits very well in either approach for elucidating the pathogenesis or finding novel drug targets for a specific subgroup of AD patients [9]. 

In this review, several mechanisms of Cu toxicity identified as contributing to AD onset and progression are briefly explained, with a focus on the involvement of microglia and astrocytes in the context of Cu imbalance playing a part in AD pathogenesis/development in a subset of AD patients.

## 2. Microglia and Astrocytes

### 2.1. Microglia and Astrocytes in Health 

Among immune cells, the CNS is primarily populated with microglia, which constitutes slightly less than 10% of CNS cells. Besides microglia, cells pertaining to other types of mononuclear phagocytes (i.e., CNS-associated macrophages) populate distinct CNS regions—meninges, choroid plexus, and perivascular spaces, from which their nomenclature arise [10]—servicing in barrier functions. Together with microglia and other non-neuronal cells, they contribute to the regionalization of the peculiar immunity, metabolism, and physiology of the CNS. In the steady state, and with the partial exception of choroid plexus macrophages, all of these CNS mononuclear phagocytes are maintained throughout life by local turnover/self-renewal [11]. While the infiltration of blood-borne monocytes and their differentiation into microglia-like cells may take place following inflammatory and disease conditions in experimental animal models and perhaps the human CNS, the in situ slow rate of proliferation provides a major source of microglia. Early migration of their precursors into the developing brain and co-habitation during CNS cell differentiation endow microglia with a well-integrated set of functions, beyond debris scavenging. This contributes to a balanced incorporation of neurons into functional circuits. First, microglia control the number of neurons interfering with both embryonic and adult neurogenesis by actively phagocytosing neuronal precursor cells [12] and by feeding neurons with soluble factors that can either support their viability, such as insulin-like growth factor 1 (IGF1)] [13], or induce cell death, such as nerve growth factor (NGF) [14]. Afterwards, during brain development, microglia prune weak synapses tagged by complement components [15] and the phagocytic receptor triggering receptor expressed on myeloid cells 2 (TREM2) [16], thus limiting excessive redundancy in neural circuits [17]. 

Astrocytes, also known as astroglia, exhibit a star-shaped morphology with several cellular processes spreading from the soma [18]. Astrocytes, the most numerous cell type in CNS, have an intimate physical association with blood vessels and single, as well as groups of, synapses in the brain. They perform a variety of functions, including axon guidance and synaptic support, regulation of extracellular concentrations of ions, neurotransmission, and control of the blood–brain barrier (BBB) and blood flow. Astrocytic terminal processes or end-feet form a lacework of fine lamellae by completely covering the outer surface of the endothelium. Astrocytic end-feet release various soluble factors, such as glial cell-line neutrophic factor (GDNF), transforming growth factor-beta (TGF-β), basic fibroblast growth factor (bFGF), and angiopoetin-1 (ANG-1), thereby regulating the process of angiogenesis and the formation of endothelial cell-to-cell junctions that preserve the structural and functional integrity of the BBB [19]. It is known that neurons and glia cooperate in the modulation of cognitive functions [20] by controlling synaptic plasticity and neurotransmission. Astrocyte dysfunction has an effect on neurotransmission and may result in different neuropsychiatric disorders, as well as neurodegenerative diseases, including AD [1,2]. 

### 2.2. Microglia and Astrocytes in Alzheimer’s Disease

Several emerging studies have suggested the involvement of glial cell deregulation as a major contributor of cognitive deficits and neurodegenerative processes seen in AD. Compelling evidence has revealed the biphasic roles of microglia in the pathogenesis of AD. According to previous literature, microglial phenotypes in AD were defined by the occurrence of specific cell surface molecules and cytokines, such as the classical activation (M1; proinflammatory and cytotoxic) phenotype and the alternative activated (M2; participates in subsiding inflammation) phenotype. Impaired microglial polarization with excessive activation of the M1 phenotype and dysfunction of the M2 phenotype significantly facilitates AD. However, the concept of a microglial phenotype has been recently redefined to correctly explain the complex physiology of microglial cells. In a healthy brain, an exclusive homeostatic molecular and functional signature (M0) [21] is present and is firmly controlled by transforming growth factor β (TGFβ) signaling [21]. In contrast, microglia transits from a homeostatic molecular signature [22] to a chronically inflammatory phenotype [23] over the course of the disease.

Recent single-cell RNA sequencing studies on the human AD brain and on a murine model of AD that expresses five human familial AD mutations (5XFAD) have observed a unique “neurodegenerative” phenotype or disease-associated microglia (DAM). This “neurodegenerative” phenotype is shared by AD, amyotrophic lateral sclerosis, frontotemporal dementia, and aging [24,25,26,27]. This “neurodegenerative” phenotype is triggered by the activation of TREM2-APOE signaling, which suppresses the unique homeostatic molecular and functional microglial phenotype (M0) [25,28]. DAMs are characterized by a downregulation of key homeostatic genes (e.g., *P2ry12*, *Tmem119*, *Cx3cr1, Gpr34*, *Csf1r*, *Hexb*, and *Mertk*) [25,26] with a concomitant upregulation of selective inflammatory genes, including *Spp1*, *Itgax*, *Lgals3*, *Axl*, *Clec7a*, *Ccl2*, and *Apoe* [25]. DAMs have been primarily recognized in 5XFAD, and their characteristics have been confirmed in other amyloid-β (Aβ) AD mouse models [24,25,29] and tauopathy models [24,30]. In 5XFAD and APP/PS1 models of AD, the co-localization of DAM with Aβ plaques was observed in the cortex [26,31]. The analysis of microglia from these AD models deficient in TREM2 revealed the two stages of DAM: Stage 1 (TREM2-independent) and Stage 2 (TREM2-dependent). Based on these recent findings, it was demonstrated that three microglia subtypes, including homeostatic microglia and Stage 1 and Stage 2 DAM, were present in a mouse AD model [26]. 

In vivo and in vitro studies have proposed several mechanisms for understanding the role of microglial activation in response to neuropathological conditions [32]. During AD progression, the microglia may interact with Aβ peptides and neurofibrillary tau tangles (NFTs) through different receptors, including the receptor for advanced glycation end products (RAGEs), G protein-coupled receptors formyl peptide receptor 2, chemokine-like receptor 1, TLRs 2/4, the CD14 co-receptor, and the α6β1 integrin [32]. This association, in turn, results in a sequential overactivation of inflammatory mediators, such as inflammatory cytokines, complement components, chemokines, and oxidative stress, which is suggestive of activated microglia as a major source of neuroinflammation during AD pathogenesis. Murine studies have also indicated that mutation in the TREM2 gene, expressed by microglia in the CNS, interferes with Aβ clearance and increases tau hyperphosphorylation due to the reduced phagocytic activity of microglia cells [33,34]. The role of microglia in tau pathology is highly complex: microglial cells sequester tau for degradation but do it inefficiently and, in turn, release tau that stimulates aggregation in recipient cells [35]. Notably, a recent study found that amyloid plaques associated with hyper phagocytic DAM can also exacerbate the propagation of pathologic tau through extracellular vesicle secretion in a humanized APP mouse model [36]. Moreover, activated microglia can also cause synapse loss, likely via a complement-dependent mechanism and, therefore, also aggravate tau pathology and release inflammatory factors that can damage neurons directly or indirectly by the induction of neurotoxic astrocytes [37]. For instance, the genetic deletion of the complement factors C1q and complement component 3 (C3) or the microglial complement receptor CR3 decreases the early synapse loss and the number of activated microglia, which, by provoking the induction of phagocytic microglia, is suggestive of complement activation as an early mediator for plaque-associated synapse loss in AD brains [38,39,40]. 

Conversely, microglia can also exert a neuroprotective role by generating anti-Aβ antibodies and enhancing the clearance of amyloid plaques and tau seeding. Furthermore, Aβ has been implicated in the microglia free-radical production in response to Aβ [41]. Microglia efficiently take up Aβ aggregates when Aβ forms a complex with lipoproteins, such as low-density lipoprotein (LDL), apolipoprotein E (apoE), and clusterin/apolipoprotein J (CLU/apoJ) [42,43]. The interaction between microglia and the Aβ/amyloid precursor protein (APP) through specific pattern recognition receptors (PRRs), including CD14, CD36, and TLRs, on microglial surfaces activates Aβ clearance by inducing microglial phagocytosis [44]. In addition, lipopolysaccharides (LPSs), inducers of inflammation, have been observed to activate microglia for the potentiation of Aβ degradation [45]. However, previous literature has suggested an opposite role for LPSs: exacerbating tau pathology and inhibiting or unaffecting Aβ clearance. This makes the contribution of LPSs in Aβ clearance questionable [46,47]. Additionally, LPSs and other bacterial products, such as *E. coli* K99 pili protein, have also been detected in AD specimens [48,49]. Notably, LPSs colocalize with Aβ_1–40/42_ in amyloid plaques and around vessels in AD brains [48]. Cathepsin B (Cat B), a lysosomal cysteine protease, also participates in microglia-mediated Aβ clearance in the early stages of AD [50]. A recent study has elucidated that TREM2-dependent activation of the DAM phenotype mitigated Aβ-induced pathological tau seeding and spread in a mice model of AD [36]; however, the ablation of both TREM2KO and microglia reversed the effect by dramatically increasing tau propagation and spreading around plaques [36]. 

Recent studies have also shown evidence for the existence of a causal relationship between astrocytes and neurodegeneration. Astrocytes are also known to elicit an effect that may be neuroprotective or deleterious in the AD. Astrocytes can become reactive under certain acute or chronic stressful conditions and correspond to “A1” proinflammatory and “A2” anti-inflammatory, respectively [51]. In vitro and in vivo studies have reported the accumulation of reactive astrocytosis (the A1 phenotype), particularly around amyloid plaques encircling Aβ deposits in AD brains [52]. The activation of astrocytes by Aβ or from damage or injury, or activated microglia, can trigger the overproduction of inflammatory cytokines, including interlukin-1 (IL-1), C1q, and TNF-α, which results in the generation of oxidative stress and, subsequently, promotes neurodegenerative processes in AD [53].

The mechanisms related to the interaction of astrocytes with Aβ are not clearly known; however, astrocytes are known to express a variety of receptors, such as the RAGE, lipoprotein receptor-related proteins (LRPs), membrane-associated proteoglycans, and scavenger receptor-like receptors, which can identify and bind to Aβ (reviewed in Reference [54]). Moreover, Aβ aggregates can also induce the production of chemotactic molecules (monocyte chemoattractant protein-1 (MCP-1)), which favor the recruitment of astrocytes to the site of injury [55]. According to previous research, Aβ aggregates can also support the inflammatory processes induced by astrocytes [56]. Aβ stimulates NF-κB and complement signaling in astrocytes, which may induce the synthesis of inflammatory mediators by astrocytes and impair synaptic density and dendritic morphology [57,58], responsible for neurodegenerative changes in AD. Astrocytes cause nitric oxide (NO)-mediated neurotoxicity by increasing the expression of inducible nitric oxide synthase (iNOS) and contribute to sustained neuroinflammation [59]. Additionally, the astrocyte A1 phenotype is known to cause nerve damage by enhancing the classical C3 factor [53]. Interestingly, these could also drive the production of Aβ under certain inflammatory conditions, such as TGF-β1 alone [60], or IFN-γ in combination with TNF-α [61,62] or IL-1β [62]. 

Astrocytes can also engulf partly digested Aβ, which lead to astrocytic defects and neuronal apoptosis [63]. There is also evidence that reactive astrocytes display an increase in the level of APP, β-secretase (BACE1), and γ-secretase, which assemble the essential machinery accountable for forming Aβ peptides and their aggregates [61,64]. Complicating the matter, they also blunt the plaque buildup by helping in clearing the Aβ peptide, as shown by in vitro and in situ studies [55]. Beyond the role of astrocytes in Aβ aggregation, the phenotype A2 is also involved in the clearance of Aβ, which is indicative of the neuroprotective role in the neurodegenerative processes of AD [55]. In fact, AD mouse models demonstrate reactive astrogliosis even before the appearance of Aβ deposits and suggesting it as a very early event [65]. Strikingly, these cells are also closely involved in Aβ catabolism [64]. Human studies involving prodromal AD subjects corroborate findings in animal studies, wherein monoamine oxidase activity was significantly higher and indicated astrogliosis [66]. On a similar line but an extended approach, the local ablation of astrocytes resulted in neurodegeneration in specific regions of animal models, which may be partly due to a decrease in the neuroprotective activity of astrocytes and increased excitotoxicity [67]. 

It is believed that astrocyte-induced Aβ clearance may be stimulated by an increment in the expression of proteases, such as neprilysin, insulin-degrading enzyme, endothelin-converting enzyme (ECE), and metalloproteases [68,69,70,71]. There are also several complementary mechanisms related to the secretion of extracellular chaperones by astrocytes, including ApoE, apolipoprotein J (Apo J)/clusterin, α2-macroglobulin (α2-M), and α1-21 antichymotrypsin (ACT), which participate in Aβ clearance across the BBB [54,72,73,74]. The A2 phenotype displays protective features by upregulating several neurotrophic factors, such as S100 calcium-binding protein A10 (S100A10) and brain-derived neurotrophic factor [51,53]. Interestingly, astrocytes form a structural protective barrier between amyloid deposits and neurons, which preserve neural tissue and restrict the deleterious effects of inflammation in AD cases [55]. Beyond their role in removing or degrading Aβ, astrocytes can also release many trophic factors that may exert a biphasic effect in AD. For example, astrocytes maintain neuronal function and cognitive performance by secreting GDNF in aged rats [75], whereas overexpression of NGF can cause neurotoxicity, as well as damage to hippocampal neurons [76]. The crosstalk between microglia and astrocytes in the neuroinflammatory process is increasingly gaining attention in the field of glial research. In fact, the shift to the A1 phenotype is regulated by microglial activation and the coexistence of both A1 astrocytes, and activated inflammatory microglia has been seen in the prefrontal cortex of AD patients [77].

Moreover, in vitro experimental studies have observed that glial cells can be activated by metals, a hallmark of neuroinflammation that facilitates APP expression and insoluble Aβ formation [78]. In this context, the next section of this review highlights a synopsis of the chemical, biological, and toxicological aspects of Cu and emphasizes oxidative stress as a crucial event in facilitating its toxicity in AD [79].

## 3. Main Processes of Oxidative Stress in AD: The Linkage with Cu Imbalance

Exposure to Cu, particularly the divalent form, has been proposed to augment the risk of an onset of AD [80]. Coordination chemistry along with meta-analysis and with epidemiological and genetic studies [79,81] show involvement of Cu and non-ceruloplasmin Cu (also known as ‘free’ Cu) [82], a component of serum Cu not bound to proteins and specifically not bound to ceruloplasmin, from which its name is derived, in AD, as posited by the Cu–AD hypothesis (reviewed in Reference [83]). This hypothesis theorizes that an age-driven Cu imbalance results in a gradual shift from protein-bound metal ion pools to pools of loosely bound metal ions that drives a bloodstream non-ceruloplasmin Cu excess in AD [84]. 

This hypothesis stems from the increasing number of experimental, meta-analysis, and multifactorial studies that have delineated that chronic exposure to Cu and its imbalance is linked to accelerated cognitive decline and AD pathology in the human brain [79,85,86,87,88]. As evidenced by seminal dietary experiments [89] and confirmed in AD transgenic models [90,91,92], a paradigm of 9–12 months of exposure to 1.3 ppm Cu in drinking water unraveled the mechanism of Cu toxicity linked to AD pathology. Chronic Cu exposure (1.3 ppm Cu in drinking water) after 9 months interferes with the LRP1-mediated clearance of Aβ alterations in cortical vasculature by altering microRNA dynamics, which indicates an involvement of Cu exposure in brain vascular damage by augmenting vascular Aβ accumulation and, thereby, increasing the risk for developing cognitive decline and AD [90]. Preclinical studies clearly show how chronic Cu exposure increases non-ceruloplasmin Cu in plasma and in brain capillaries [91,93], mimicking non-ceruloplasmin Cu excess noted in AD patients [94] and, particularly, in a subset of AD patients [83], which is typified by non-ceruloplasmin Cu levels higher than 1.6 μmol/L and susceptibility to Cu disturbances on a genetic basis [81,95]. Notably, non-ceruloplasmin Cu crosses the BBB, which is exemplified by Wilson disease, the paradigmatic disorder of Cu accumulation on a monogenic basis. In AD, as well, its excess can lead to impairment in the hippocampal synaptic structure and spatial memory, as shown in preclinical studies and confirmed in AD brain specimens [90,96,97].

In the next sub-sections, we will discuss in detail how Cu can directly bind with Aβ and promote the accumulation and oligomerization of Aβ, which aggravates reactive oxygen species (ROS) generation, responsible for oxidative neuronal damage [98].

### 3.1. Oxidative Stress and the Loss of Functional Cu in a Protein-Bound Pool

In this section, we refer to functional Cu in a protein-bound pool as Cu that is structurally bound in proteins/enzymes that have a well-defined function in the cell/tissue, particularly those that play a role in the brain. This is opposite to the loosely bound, exchangeable Cu (non-ceruloplasmin Cu), which is Cu that can be exchanged among peptides, amino acids, and albumin and that does not have a specific function but can be prone to oxidative stress (reviewed in Reference [83]). There are, in fact, two oxidation states of Cu: cuprous Cu(I) and cupric Cu(II) ions. Cu is mostly transported and buffered in its Cu(I) state intracellularly, bound to, for example, metallothioneins (MTs), Cu transporter 1 (Ctr1), and ATPases ATP7A/B, and Cu(I) in the free form is dangerous. Cu(II) is functionally important in enzymes/proteins that perform a precise function in the cell/tissue. These cuproenzymes play their catalytic function by using Cu(II)/Cu(I) redox cycles. However, if Cu is not employed for a catalytic function, it can enter Cu(II)/Cu(I) Fenton-type redox cycles and produce ROSs in a continuous manner. Cu(II) in non-ceruloplasmin Cu can increase the amount of the Cu redox pair that commonly participates in aggravating oxidative stress via Fenton-type chemistry: Cu(II) in the presence of a superoxide anion radical or biological reductants can be reduced to Cu(I), which is capable of decomposing hydrogen peroxide (H_2_O_2_) to form reactive hydroxyl radicals (•OH and OH–) via the Fenton-type reaction (refer to Reaction 1 and 2) [83,99].

Cu(II) + O_2_− • → Cu(I) + O_2_, (1)

Cu(I) + H_2_O_2_ → Cu(II) + •OH + OH–(Fenton-type reaction). (2)

Oxidative stress via Fenton-type chemistry, driving ROS formation, including the highly reactive hydroxyl radical (HO•), is a hallmark of both AD [100] and normal aging of the human brain [101]. In fact, Cu dyshomeostasis has been observed in AD and facilitates Aβ oligomer formation and amyloid deposition, which, in turn, provoke oxidative damage by Fenton-type reactions [102]. The direct interaction of redox Cu ions with the Aβ peptide is one of the primary lesions in AD. Particularly, Cu(II) ions interact with Aβ at conserved histidine residues of Positions 6, 13, and 14 for metal coordination to engender di-tyrosine cross-linking between two Aβ peptides, which promotes the irreversible oligomerization of Aβ [103]. These metal ions are reduced by Aβ from Cu(II) to Cu(I) with a concomitant generation of ROSs, hydrogen peroxide (H_2_O_2_), and HO•, which lead to the formation of proapoptotic lipid peroxidation products, such as 4-hydroxynonenal (HNE) [104].

Moreover, elevated Cu promotes ROS formation and its cytotoxic effects by reducing the level of glutathione (GSH) (a substrate for enzymes that removes ROSs). The probable reason for the reduced GSH level in the blood of Cu-exposed animals may be related to the interaction between Cu and GSH, which forms a Cu(I)-[GSH] complex that is responsible for converting molecular oxygen (O_2_) into superoxide [105]. GSH depletion has been pointed out as the main driver of ferroptosis in AD, which is a special modality of cell death determined by an iron-dependent phospholipid peroxidation [106]. The drop in GSH provides a link between Cu imbalance and iron dysmetabolism tied to AD risk and progression [107]. The expression of Cu-dependent enzymes, such as Cu/Zn SOD1 and antioxidant protein 1 (ATOX1), was markedly reduced in multiple microarray studies of AD patients, reinforcing the notion that a lack of Cu, by reducing host antioxidant defenses, plays a pivotal role in AD pathology [108,109].

Consistently, chronic exposure of 0.13 ppm Cu in drinking water, along with spatial memory impairment, has caused variation in the expression of 31 hippocampal mitochondrial proteins (15 increased and 16 decreased) and 46 hippocampal nuclear proteins (18 increased and 28, including synapsin-2 and SOD1, decreased) in Cu-treated versus untreated mice [109]. In addition to the antioxidant role, SOD1 also exerts anti-inflammatory functions. In concordance with this notion, reduced expression of SOD1 can exacerbate ROS accumulation and chronic neuroinflammation [110]. These processes can produce a loss of functional Cu in protein-bound pools that can reduce energy production by CCO and defense against oxidative stress by SOD1, which are central components for brain function and aging [111]. 

The greater frequency with late onset AD subjects has been reported with inherited mutations of mitochondrial CCO genes [112], which is suggestive of impaired CCO activity as a risk factor for AD. Furthermore, the enzymatic activity of SOD1 has been observed to be impaired in AD brains [113] and in transgenic APP mice [114], which may be due to a decline in occupancy at the active site of Cu. Ceruloplasmin has also been observed to be lower in the brain of AD patients [115]. In fact, ceruloplasmin inhibits iron (Fe)-mediated oxidative alterations by oxidizing Fe (II) to Fe (III) [116], which is indicative of protective effects against the onset of AD. Thus, depletion of Cu-bound ceruloplasmin can trigger oxidative stress induced alterations in AD brains. 

The findings sustaining a gain in the toxic function of increased labile Cu in the synaptic cleft reported in Section 3.4 and those regarding the loss of functional Cu in protein-bound pools reported in this section fit well with the Cu–AD hypothesis (reviewed in Reference [84]).

### 3.2. Abnormal Cu Handling in Mitochondria

Cu is critical for the normal functioning of mitochondria (during mitochondrial respiration), and any functional Cu loss may impair the electron transport chain function and deplete neuronal energy. This event may further trigger a leakage of ROSs from electron chain transporters followed by impairment in the mitochondrial transmembrane potential and ROS-induced apoptosis [117].

Cu acts as a cofactor for enzyme CCO, which catalyzes the reduction of molecular oxygen (O_2_) to water (H_2_O). This results in the generation of the electrical gradient, which is used in ATP synthesis by mitochondria [118]. Cu concentration within mitochondria is controlled by Cu-transporting ATP7B, a Cu pump that may be involved in the export of Cu from mitochondria. CCO Cu chaperone 17 (Cox17) directs the Cu in both the cytosol, as well as intermembrane space, of mitochondria. Three Cu ions are present in the active assembled form of CCO, Complex IV of the electron transport chain. Hence, inefficient Cu transport to mitochondria may result in a poor assembly of individual CCO subunits into the functional holoenzyme [119,120]. Consequently, the accumulation of electrons upstream and leakage from Complexes I and III can generate ROSs by reducing oxygen to the superoxide, which causes the release of CCO into the cytosol to activate caspase-9 for the execution of the apoptotic pathway. Furthermore, any mutation of Cox 17 might also affect the function of mitochondrial respiration [119]. Any of such defects in CCO biogenesis due to the loss of Cu impair energy production and mitochondrial dysfunction, which leads to AD and other neurodegenerative diseases [121]. Of note, CCO is expressed in 2/3 cells of the molecular layer of the dental gyrus in the hippocampus [122]. A number of studies have reported a decrease in CCO expression [110,123] or activity [124] and impaired energy metabolism in an AD patient’s brain [125,126,127]. It is well evident that Cu is required by various important enzymes for their catalytic activity, which is altered in AD brains.

### 3.3. Accumulation of the Amyloid Peptide, Protein Misfolding, and Plaque Formation 

In AD, APP undergoes fragmentation and yields peptides of variable length, which are known as amyloids. It precipitates and results in the production of both extracellular Aβ plaques containing oxidized and metal-bound Aβ peptides in the brain of AD patients, known as senile plaques and intracellular NFTs [128]. Cu can drive the intracellular aggregation of hyperphosphorylated β-sheet-rich (tubulin-associated unit) tau due to the presence of Cu-binding sites on these proteins [128,129]. It is pertinent to note that APP expression acts as a key regulator of neuronal Cu homeostasis [130]. APP has a specific Cu binding domain (CBD) located in the N-terminal cysteine-rich region, and this domain can strongly coordinate Cu(II). A CBD can influence Cu homeostasis and Aβ production. Importantly, Cu binding with the Cu binding domain of APP can greatly favor the reduction of Cu(II) to Cu(I), which, in turn, triggers the catalytic generation of ROSs via Fenton-type chemistry and Aβ aggregation [131]. It has been observed that the binding of Cu with Aβ accelerates the rate of spontaneous Aβ accumulation in contrast to the absence of metal ions [132,133]. Cu(II) shows a sub-nanomolar affinity for Aβ _(1–40)_ and Aβ _(1–42)_ (for monomers and fibers) [134] and a stronger femtomolar affinity for truncated forms of Aβ _(11–40)_ and Aβ _(4–40)_, which are capable of affecting fiber assembly [135]. On the contrary, information regarding the affinity of Cu(I) for Aβ is scarce but has been reported for Aβ _(1–16)_ [136]. As previously discussed, the disruption of Cu(II) homeostasis in the brain is a prominent characteristic of AD, which is evidenced from the presence of a 26-fold greater concentration of Cu(II) ions within senile plaques of AD patients, compared with the extracellular space of healthy individuals [137]. It is noteworthy that Cu in more damaged AD brain areas likely decreases in association with a consistent decrement of Cu within neuronal cells [96,138], while labile Cu has a 20% increase [68]. Therefore, Aβ plaques are regarded as a ‘metal sink’ [139].

Metal ions have been reported to alter the kinetics and thermodynamics of the form and structure of the aggregates. Both in vitro and in vivo studies have suggested that Cu(II) ions play an important role for Aβ42 aggregation via the amyloid cascade process, involving a transition of a normal soluble amyloid peptide to an aggregated and fibrous form. Cu(II) promotes different types of aggregation states by depending upon multiple factors, including pH, protein concentration, metal-protein ratio, temperature, buffer conditions, and stirring during incubation. Cu ions bind rapidly to coordination sites of histidine, with high affinity in a monomeric Aβ [140], thereby stabilizing Aβ aggregates [141]. Indeed, the interaction between Cu ions and monomeric Aβ potentiates the precipitation and accumulation of insoluble peptide aggregates in the amyloid plaque [142]. Notably, recent human-based evidence using synchrotron X-ray spectromicroscopy has observed the presence of newly discovered elemental metallic Cu (0) (zero-oxidation state) within amyloid plaque cores isolated from the grey matter of AD brains, which is strongly suggestive of the role of metals in the etiology of AD [85]. Furthermore, Cu exposure may also lead to modifications in the distribution, activity, and levels of enzymes, such as BACE1 [143], α-secretase [144], and presenilins, which cause the processing of APP to amplify the generation of Aβ. 

### 3.4. Increased Labile Cu in the Synaptic Cleft 

Cu in the synaptic cleft can directly or indirectly regulate the activity of neurotransmitter receptors, such as N-methyl-D-aspartate (NMDA), α-amino-3-hydroxy-5-methyl-4-isoxazolepropionic acid (AMPA), gamma-aminobutyric acid (GABA), and purinoreceptor (P2X) receptors, thus affecting neuronal excitability [145,146]. In contrast, neurotransmission can also influence trafficking and the delivery of Cu in neuronal cells. Moreover, Cu has also been reported to modulate the trafficking of synaptic vesicles and proteins [109] and the interaction of proteins that are connected with neurodegenerative diseases, such as APP, the prion protein (PrP), and α-synuclein (α-syn). At the synapse, APP can facilitate the reduction of Cu(II) to Cu(I) and, thus, enable the Cu transporter 1 (CTR1) function, and ATP7A/B can promote Cu translocation into vesicles that can then be released at the synaptic cleft. CTR1 transfers Cu(I) to Cu-chaperone for SOD1 (CCS) and metallothionein (MT), and CCS delivers Cu(I) to SOD1. The catalytic reduction of molecular oxygen (O_2_) to water (H_2_O) by cytochrome c oxidase (CCO) generates an electrical gradient used by mitochondria to create ATP [147]. Excess Cu ions are released at glutamatergic synapses in the form of labile Cu not bound to proteins during transmission in a recycling process regulated by PrP or from the postsynaptic terminal upon stimulation of the N-methyl-D-aspartate (NMDA) receptor downregulating the NMDA receptor activity [66,147]. Labile Cu can disrupt the APP capacity to reduce Cu(II) to Cu(I) and enable Cu transporter 1 (CTR1) to facilitate the ATP7B translocation of Cu into synaptic vesicles. This phenomenon facilitates Cu–Aβ formation and oxidative stress as depicted above. Conversely, it has been reported that increased labile Cu in the cleft may enhance synaptic activity, which might, in turn, be either toxic or non-toxic [148]. However, processes of synaptic activity modulation must be strictly regulated. In the case of AD, our hypothesis is that these processes are disturbed by labile Cu excess (for a specialized review, refer to Reference [84]).

### 3.5. Advanced Glycation End-Products

Advanced glycation end-product (AGE) formation is a known feature of AD [120]. Cu accelerates the AGE formation, which also promotes protein glycoxidation [149]. AGEs damage the arterial wall in diabetes and facilitate a progressive Cu-trapping potential [150]. In AD, insoluble Aβ plaques contain high levels of AGEs and transition metal ions, such as Cu, Zn, and Fe [151]. AGEs are formed by non-enzymatic reactions of lysine and arginine residues of proteins, with reducing sugars or dicarbonyl compounds, such as methylglyoxal (MG) and glyoxal formed during defective glucose metabolism or oxidative stress [152]. Aβ glycation by sugars generates the free radical superoxide anion (O_2_^−^), which, upon reacting with Cu(II), converts to HO• via a Fenton-type reaction [153]. 

Mechanistically, AGEs exacerbate neurotoxicity by upregulating receptors of AGEs, namely RAGE expression and glycogen synthase kinase-3 (GSK-3) activation. The overexpression of RAGEs has been found in AD brains and specifically acts as a binding site for Aβ at the plasma membrane of different cells, including neurons, microglial cells, and endothelial cells, of the vessel wall [154]. The interaction of AGEs with surface RAGEs of neural cells in the brain [154] perturbs cellular functions by stimulating Aβ-mediated oxidative stress, NF-κB [155], the neuronal expression of macrophage colony-stimulating factor [156], and cell death [157]. Consistently, RAGE-mediated signaling induced by glycated Aβ has been reported to be responsible for learning/memory deficit in transgenic models of AD [158]. Aβ-AGEs may also cause memory and synapse impairment by activating RAGE-dependent GSK-3 induction, which is evident from the pathological changes of Aβ-AGEs after exposure to the GSK-3 inhibitor. RAGEs can also aggravate the neurotoxicity of Aβ-AGEs by mediating the intraneuronal transport of Aβ across the BBB, thereby causing their deposition in the brain, which is responsible for the age-related memory decline in AD models [159]. 

## 4. Altered Cu Homeostasis in Microglia and Astrocyte in AD

Trace metal concentrations in different brain areas are deregulated during aging and inflammation. This particularly occurs at the choroid plexus due to a perturbed BBB. Microglia and astrocytes can restrain metals and protect neurons from metal toxicity [160,161,162]. 

### 4.1. Microglia Role in Cu Imbalance Linked to AD

As summarized above, biometal dyshomeostasis plays an important role in the pathogenesis of AD. In this section, we will discuss how Cu exposure can also prompt the activation of microglia-mediated neuroinflammation and trigger neuronal death and memory deficits [163]. There is scanty knowledge available so far regarding the dual protective or detrimental role of microglial metal homeostasis in pathogenesis of AD brains. In relation to neuroinflammation, the Cu–Aβ complexes augment the release of TNF-α, as well as IL-1β, and affect the phagocytic behavior of the murine BV2-microglia phenotype with a concomitant downregulation of LRP-1 expression in human primary microvascular endothelial cells [90,91,162]. The decreased expression of LRP-1 can further interfere with the transcytotic clearance of Aβ, thereby aggravating neuroinflammation [164,165]. 

Consistent with these findings, chronic Cu exposure has been demonstrated to suppress LRP-1 in the endothelial cortical vasculature of C57BL6 mice treated with Cu (0.13 mg/L) [91]. Moreover, trace amounts of Cu (0.21 ppm) have been reported to induce Aβ deposits and their toxic effects, which lead to the activation of microglia and stimulate the toxicity of microglia to neighboring neurons via TNF-mediated signaling in a murine model co-treated with cholesterol and Cu [166]. Aβ:Cu(II) complexes are related to neuroinflammation via involvement of TNF-α signaling accompanied by the oxidative-stress-induced apoptosis (caspase-3 activation). At the same time, Cu-induced TNF-α inflammatory signaling promotes the degradation of IκB, which implies the elevated expression of NF-κB in the hippocampus and cerebral cortex. These findings suggest that inflammatory responses in activated microglial and neighboring neuronal cells aggravate the neurotoxicity that ultimately leads to the neurodegenerative process [166].

Hu and colleagues reported that subneurotoxic doses (20 μM) of Cu(II) trigger mitochondrial ROS—NF-κB signaling that might be responsible for the release of TNF-α and NO in a dose- and time-dependent manner in rat and murine microglial cell lines. These proinflammatory factors may act synergistically to elicit the morphological alteration of microglia from resting to active states, which would eventually lead to microglia-mediated neuroinflammation and damage [163,167]. In line with this statement, another study suggested that subneurotoxic concentrations of the Cu(II)-Aβ complex treatment (but not Aβ or Cu(II) alone; 50 μM) provoke the consequences of Aβ by switching the phagocytic to the inflammatory phenotype of microglia with a simultaneous release of TNF-α and NO [163]. The Aβ-primed microglia proinflammatory phenotype activation may involve NF-κB activation and NOX-independent mitochondrial ROS production, which can further trigger the release of proinflammatory cytokines and chemokines. In fact, Cu(II) may act as a cofactor to potentiate the effect of Aβ on microglial activation and the subsequent neurotoxicity [168]. Kitazawa et al. have reported that Cu exposure can effectively reduce the phagocytic clearance of Aβ and aggravate Aβ-induced pro-inflammatory cytokine release (IL-1β, TNF-α, and IL-6), which shows a robust impact on the downregulation of LRP1 expression, as well as impairment in microglial phagocytic activation, and, finally, leads to a vicious cycle responsible for the pathological accumulation of Aβ in the brain [164].

A recent in vivo transcriptomic analysis of microglia in neurodegeneration has highlighted that excess Cu intake through drinking water shifts the microglial phenotype towards degenerative expression by increasing the translation of degenerative genes and repressing homeostatic genes, which thereby accelerates cognitive decline and may illustrate one of the major mechanisms linking Cu dyshomeostasis to the AD pathology [169]. In vivo and in vitro analysis have suggested that Cu(II) diminishes the microglia-mediated clearance of Aβ 1–42 via modulating the mammalian target of rapamycin (mTOR) transcription factor EB (TFEB) axis attributable for the suppression or impairment of lysosomal biogenesis and autophagic flux. Additionally, Cu(II) negatively targets the expression of TFEB genes, including LAMP 1/2 and Cat B, which are responsible for maintaining lysosomal integrity and Aβ clearance, respectively [170]. This effect of Cu(II) implies one of the novel mechanisms for the Cu(II)-induced neurodegeneration in AD [170]. 

In aging, interferon γ-activated microglial cells have been connected with high Cu uptake mediated by an enhanced expression of a Cu importer, specifically CTR1 in the choroid plexus [161]. Similarly, Ashraf et al. have observed the enrichment of Cu in aging ventricles, likely due to inflammation, indicating a compromised barrier system. Additionally, age-related glial dystrophy/senescence has also been supposed to perturb metal homeostasis due to oxidative stress and, thus, amplify the risk of neurodegenerative diseases [171]. Notably, an in vitro study revealed that increased concentrations of ceruloplasmin in AD patients can potentiate the proinflammatory activation of microglial cells by upregulating NO release and the mRNA profile of encoding interleukins and enzymes, such as cyclooxygenase-2 (COX-2) or NADPH oxidase [172].

Alternatively, the intracellular sequestration of Cu(II) has been reported to activate the neuroprotective phenotype of microglia, which may further abrogate Aβ plaque formation [173]. Mechanistically, activated microglia surrounding Aβ plaques upregulate the expression of ATP7A, which may favor overall Cu(II) uptake by augmenting CTR1 Cu importer expression, as observed in a TgCRND8AD mice model [161]. Consistent with this, the upregulation of ATP7A expression was also observed in BV-2 microglial cells treated with proinflammatory agents (such as IFN-γ), which elevated Cu uptake and CTR1 expression in AD patients [161]. These observations revealed that Cu uptake via CTR1 and sequestration via ATP7A can alter the Cu homeostasis in activated microglial cells at sites of amyloidogenesis in AD brains. Hence, the uptake and sequestration of Cu(II) by microglia confers one of the key neuroprotective mechanisms in AD, which limits the labile extracellular Cu requisite for Aβ aggregation and plaque formation [161].

On the contrary, an in vivo study has revealed that the transfection of fibroblast cell lines with ATP7A can cause the depletion of intracellular Cu, which, by downregulating APP expression, inhibits Aβ production [174]. However, there is also a contradictory report stating that the deficiency of Cu can lead to aberrant microglial activation and subsequently contribute to neurodegenerative diseases [175]. This effect suggests that Cu plays a key role in the maintenance of microglial homeostasis, as well as in AD, and its role/concentration changes in different regions of brain/glial cells also depend upon the disease state, i.e., during early phase, the middle stage, and the final, full blown AD stage, or in some specific subset of AD patients who are genetically more susceptible to Cu imbalance.

Moreover, the spectrum of the microglial phenotype depends upon the redox capability of Cu. This is evident from the role of the Cu ion (I) in polarizing activated microglia from the proinflammatory M1 phenotype to the anti-inflammatory M2 phenotype by inhibiting NO production and regulating S-nitrosothiol signaling [176,177]. This observation highlights how a Cu valence state (Cu(I) and Cu(II)) may act as a key factor in the maintenance of the microglial cell phenotype. Thus, neurotoxicity may depend on their different redox capabilities. According to Du et al., Cu(I) decreases Aβ42 aggregation and, in turn, hampers ROS production as compared to Cu(II), which is indicative of decreased neurotoxicity [178]. On the other hand, Cu(II) can predominately bind monomeric Aβ peptides (1:1 stoichiometry) at physiological pH and lead to a Cu(II)–Aβ complex formation [179]. This phenomenon may explain the different roles of microglia in neurotoxicity mediated by Cu(I) and Cu(II) during the pathogenesis of AD.

Thus, it is mandatory to understand the exact role of Cu homeostasis in the regulation of immune responses in the brain, particularly its role in microglia-mediated neurotrophic or neurotoxic effects in AD, which may provide for a novel therapeutic strategy for AD.

### 4.2. Aberrant Astrocyte Behavior and Excess Labile Cu

Astrocytes are known to maintain nervous tissue by regulating extracellular ion homeostasis, synaptic transmission, and the supply of metabolites [180]. Additionally, astrocytes are well known regulators of the homeostasis of redox-active metals, specifically Cu in the brain by influencing Cu uptake and export [181,182]. Previous studies have reported that cultured astrocytes are able to efficiently store Cu (apparent Km ≈ 10 μM for Cu uptake) [183,184,185,186]. Cultured astrocytes can accumulate Cu via CTR1-dependent and -independent mechanisms that may include divalent metal transporter 1 (DMT1) and members of the Zrt/IRT-like protein (known as ZIP) family [182,184]. An ecto-cuprireductase and/or astrocyte-derived ascorbate can mediate the reduction of extracellular Cu(II) to Cu(I) for astrocytic uptake by Ctr1 and DMT1. Interestingly, astrocytes are believed to be equipped with machinery that can deal with even higher amounts of Cu and, hence, be exceptionally resistant to Cu-mediated toxicity [187,188]. A possible reason is that astrocytes buffer excess Cu by increasing cellular tripeptide GSH and metallothioneins (MTs)—a family of metal-binding cysteine rich proteins [182,189]. Moreover, GSH also participates in the cellular Cu transport [190]. During Cu overload, excess Cu binds with GSH and MTs in astrocytes and, thereby, prevents Cu-induced neurotoxicity. Additionally, Cu is shuttled to target proteins by specific Cu chaperons, e.g., CCS to SOD1, COX17 to SCO1/2, COX11 to CCO, and ATOX1 to ATP7A. Astrocytes export Cu by expressing ATP7A, which undergoes trafficking between the *trans*-Golgi network and vesicular structures [182]. Prion protein has also been suggested to mediate Cu uptake or export in astrocytes [183].

Astrocytes, the Cu depots of the brain [5], play a paramount role in the homeostasis of the redox-active metal Cu, which upon dyshomeostasis is detrimental to cell functioning and survival [9]. Cu levels beyond the handling capacity of astrocytes may initially result in a cascade of protective events to reduce labile Cu neurotoxicity, thus activating astroglia, astrocyte hypertrophy, and related events [5,191,192]. Previous in vivo studies observed that excess Cu can decrease the cell viability in cultured astrocytes [187,193]. The toxic mode of action of Cu was observed with a significant increase in hydroperoxide levels due to the valency shift from the Cu(II) to Cu(I) state, which suggests a critical role of oxidative stress in the mode of action of Cu. Excess amounts of CuSO_4_ induce mitochondrial permeability transitions (mPTs), which are evident from a significant dissipation of the mitochondrial membrane potential (ΔΨm) and death at 48 h in cultured astrocytes. However, pre-treatment with antioxidants may reverse the effect of Cu-induced mPTs and the oxidative death of astrocytes. These observations support that oxidative stress and mitochondrial dysfunction are involved in Cu toxicity [187,194]. In agreement with this, Cu at subcytotoxic concentrations exerts cytotoxic potential (EC_30_: 250 µM) by impairing mitochondrial function specifically affecting ΔΨm, which eventually triggers ROS production, suggesting an involvement of mitochondria as a potential target organelle accountable for the toxic effects of Cu in a human astrocytic cell model [189]. Impaired CNS metal homeostasis leads to ROS production at affected sites depending on the severity/extent, which may cause irreversible damage and with time lead to neurodegeneration.

Consistent with the Cu–AD hypothesis [84], AD patients exhibit values of non-ceruloplasmin Cu that are commonly found in Wilson disease, the paradigmatic disorder of Cu accumulation or toxicosis caused by mutations in the *ATP7B* gene. In Wilson’s disease, astrocytes likely accumulate large amounts of excess Cu present in the brain, protecting neurons from Cu toxicity. This ability appears to be related with the upregulation of GSH and MTs [182]. The depletion in GSH levels is a feature of the aging brain and might be associated with neurodegenerative disease progression, including impairments in cognitive function and ferroptosis in AD [108,195]. Observations on AD patients have shown that around 3% of non-ceruloplasmin Cu can cross the BBB [196]. This provides some hints about the potential mechanism of Cu imbalance occurring with aging, and non-ceruloplasmin Cu, if it continues to rise unchecked, will disturb labile Cu balance in the interstitial space around neurons and glial cells, which might further trigger protective reversible reactive astrogliosis events, such as ischemic preconditioning or ischemic tolerance [197], which, in turn, may result in irreversible neuroinflammatory response, which can be detrimental to neuronal health. As shown previously, higher serum non-ceruloplasmin Cu levels are associated with clinical deficits that are features of an AD picture, including a lower mini-mental state examination (MMSE) score [198] and peculiar neurophysiological and brain anatomical damage (reviewed in Reference [199]), even though the exact mechanism needs to be elucidated. Redox active non-ceruloplasmin Cu in the context of a disturbed buffering capacity of astrocytes might, thus, contribute to AD neurodegeneration mirroring Wilson’s disease, and this provides for a window of opportunity to reverse this damaging process by chelating excess labile Cu [191,192].

In pathological conditions, such as AD and other neurodegenerative diseases, any disturbance in the disposition of Cu has been reported to lead to an impaired trafficking of Cu from astrocytes to neurons [182]. This is likely due to the enrichment of Cu chaperone proteins in astrocytes. Cu can especially generate HO• in astrocytes due to the abundance of Cu carrier protein-chaperones [186]. This is evident from the intracellular Cu that augments the expression of two markers for endoplasmic reticulum stress response (GRP78 and GRP94 protein), which are involved in oxidative stress response in lead-exposed astrocytes [186]. 

Another line of research proposes that astrocytes participate in a neurotrophic, as well as neuroprotective, role, and the inappropriate function of these cells may lead to an onset of neurodegenerative diseases [200]. Meanwhile, astrocytes can transform from an acute to a chronically active ‘proinflammatory’ phenotype by the dysregulation of calcineurin/nuclear factor of activated T-cell (CN/NFAT) signaling [201], which perpetuates neuroinflammatory signaling cascades and participates in the progression of neurodegenerative diseases. Indeed, activated astrocytes appear as an early hallmark of AD and started consistently increasing with the development of clinical and pathological manifestations [202]. As discussed in detail, Cu is known to accelerate the sequential cleavage of APP to produce extracellular Aβ monomers, which undergo oligomerization to form complex Aβ complexes that play a crucial role in brain inflammation. In vitro evidence has suggested that Aβ plaques and fibrils are endogenous stimuli for turning on astrogliosis or reactive astrocytosis [203]. Aβ oligomers may induce inflammatory response by affecting immune constituents of astrocytes remarkably via activating various PRRs, including TLR 2/4, RAGEs [204], and the inflammasomes [205]. The interaction between Aβ oligomers and TLR 2/4 on astrocytes can stimulate inflammatory responses, which elicit toxic proinflammatory mediators that affect neurons and, thereby, cause neuroinflammation in AD brains [203]. Several in vitro and in vivo studies, by generating a number of inflammatory mediators, including IL-1β, IL-6, MCP-1, macrophage inflammatory protein (MIP), and NO, have identified that the same phenomenon is responsible for neuroinflammation in AD [206,207,208].

It is not surprising that astrocyte deterioration causes neurotoxicity and, thereby, leads to aging and neurodegenerative disease. Cu at a physiological concentration in complexes with the metal chelator neocuproine (NCP) may cause the apoptosis of rat cortical astrocytes by inducing oxidative neurotoxicity. In fact, the NCP facilitates intracellular Cu uptake and, thereby, provokes an increase in ROS production, the dissipation of ΔΨm, and the depletion of GSH and ATP. Consequently, this results in the activation of downstream molecules, such as c-Jun N-terminal kinase (JNK), as well as caspase-3, and, ultimately, leads to PARP degradation [188]. The same group has also observed that the PDTC-Cu(II) complex induces astrocyte dysfunction through an apoptotic process with a subsequent activation of oxidative stress and JNK [209].

Griffin and colleagues have described that the long-term stimulation of these mediators can activate a chronic “cytokine cycle,” which is harmful in affecting disease progression [210]. Astrocytes express RAGEs that function as PRRs for Aβ oligomers induced by Cu accumulation [204]. The Aβ binding to RAGEs causes a proinflammatory reaction, which may be mediated through the NF-κB transcription pathway. Additionally, RAGEs are also involved in accelerating Aβ transport from plasma to the CNS, which suggests their involvement in AD pathogenesis [211]. Askarova et al. have revealed that the Aβ_42_–RAGE interaction can activate the assembly of the NADPH oxidase complex, which further leads to the induction of ROS generation, the mitogen-activated protein kinase/extracellular signal-regulated kinase 1/2 (MAPK/ERK) pathway, and the phosphorylation of cytosolic phospholipase A_2_ (cPLA_2_) in primary astrocytes [212]. Additionally, astrocytes have been reported to hinder microglia-mediated Aβ plaque clearance by the secretion of glycosaminoglycan-sensitive molecules [213,214], and this indirectly facilitates Aβ accumulation in AD brains.

On the contrary, Choo et al. have recently demonstrated that the Cu-bis (thiosemicarbazone) complex Cu^II^ (atsm) alleviates brain inflammation induced by the peripheral administration of bacterial LPS. Cu^II^(atsm) significantly reduces the secretion of NO, MCP-1, and IL-6 in astrocytes. These robust anti-inflammatory effects of Cu^II^ (atsm) may be connected with increased cellular Cu levels and MT-1 in astrocytes [215].

## 5. Conclusions

**Figure 1:**
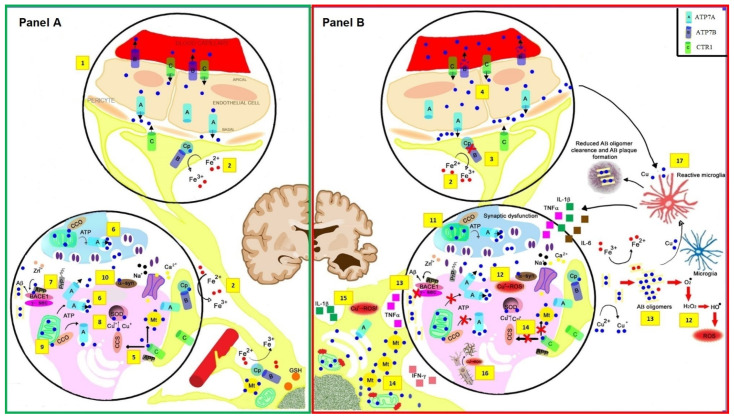
Model of copper (Cu) Physiology ((**A**) **green box**, **up**) and Pathology ((**B**) **red box**) in critical areas of the brain related to Alzheimer’s disease (AD). At the apposition of the endothelial cells composing the blood–brain barrier (BBB) and the astrocyte foot (1) Cu-related trafficking is facilitated by the CTR1 transporter (Cu shown as blue circles). The two Cu-pump proteins Cu-transporting P-type ATPase, ATP7A and ATP7B, control the metal extrusion from endothelial cells to the bloodstream (ATP7B) or the interstitial fluid (ATP7A). Inside astrocytic feet, ATP7B also promotes Cu loading into glycosylphosphatidylinositol (GPI)-linked ceruloplasmin (Cp) controlling Fe(II)/Fe(III) oxidation state (2). In the AD brain ((**B**) **red box**, **up**), excess Non-Cp Cu in the bloodstream is a source for the buildup of labile Cu^2+^ in the interstitial space, promoting ATP7A/B translocation of Cu^2+^ into vesicles of the trans-Golgi network and endoplasmic reticulum (ER). Specific variants of the ATP7B protein (for example, R832 and K952, but additional variants may exist) can negatively impact Cu loading into nascent GPI-Cp facilitating Cu accumulation inside astrocytic feet (3); mutant ATP7B can also affect Cu export from endothelial cells and contribute to Cu dysregulation (4). Model of Cu role at the glutamatergic transmission that occurs in the hippocampus at the pyramidal CA1 synapse ((**A**) **green box**, **bottom**). At the synapse, amyloid precursor protein (APP) can reduce Cu(II) to Cu(I) to enable Cu transporter 1 (CTR1) function (5), and ATP7A/B can facilitate copper translocation into vesicles which can then be released at the synaptic cleft (6). APP/Aβ system aids Cu transport at the synapses, and, in this model, beta-secretase (BACE1), as well as γ-secretase control production, of Aβ and Cu exposure, leads to increased APP expression, and Aβ monomers protect against Cu- and iron-induced toxicity, and extracellular Prion protein (PrP), α-synuclein, and APP can buffer Cu(II) within the synaptic space, where it can reach high concentrations (100–250 µmol/L) (7). CTR1 transfers Cu(I) to copper-chaperone for SOD1 (CCS) and metallothionein’s (MTs) and CCS transfers Cu(I) to SOD1 (8). The catalytic reduction of molecular oxygen (O_2_) to water (H_2_O) by cytochrome c oxidase (CCO) generates the electrical gradient used by mitochondria to create ATP (9). At the glutamatergic synaptic cleft, Cu(II) is released in a ‘free’ form either from presynaptic vesicles during transmission in a recycling process regulated by PrP or from the postsynaptic terminal upon stimulation of the N-methyl-D-aspartate (NMDA) receptor downregulating the NMDA receptor activity (10). In the AD brain ((**B**) **red box**, **bottom**), disruption of APP/Aβ system Cu transport may not restrain high Cu concentration. In turn, ROS-driven Cu^2+^ mobilization can aggravate oxidative stress and initiate Aβ oligomerization (13). Furthermore, ROS can mobilize Cu^2+^ from MT-3 (14), leading to increased intracellular toxic Cu^2+^ concentrations and mitochondrial dysfunction. Astrocyte activation occurs with release of interleukins (IL) (15). Cu reacts with tau protein contributing to neurofibrillary tangles formation via ROS production (16). Cu and ROS activate microglia that changes to reactive microglia phenotype and release IL (17). Calcium, Ca^2+^; Copper, Cu; Iron, Fe; H_2_O_2_, hydrogen peroxide; Metallothionein, Mt; Cu/Zn superoxide dismutase, SOD1; Sodium, Na+; γ-secretase, γ-sec; α-synuclein, α-syn, Zinc, Zn.

Though not exhaustive, the evidence provided shows that aberrant glial cell biology might be involved in the deprivation of the physiological metal microenvironment around neurons, which can have direct injurious effects on functioning and viability of brain neuron cells. On this basis, it is tempting to speculate that microglia and reactive astrogliosis associated with Cu ion imbalance might play a role in processes of neurodegeneration associated with dementia encompassing the energy depletion of high-energy demand neurons, oxidative stress, protein misfolding, and glycoxidation (Figure 1).

One would predict that restoring Cu physiology may be beneficial for AD in terms of the delay of disease progression by improving glial functioning, though this needs to be clinically tested. Although this review reports a number of processes of glial involvement in AD, the role of microglia and reactive astrogliosis in response to Cu toxicity is still enigmatic [216]. Knowledge on the labile Cu–glia–AD axis is still in its infancy, and further investigation is needed to determine the pathways of all players in this promising field of research.

## Data Availability

Not applicable.
